# Frequency of health care provider recommendations for HPV vaccination: a survey in three large cities in China

**DOI:** 10.3389/fpubh.2023.1203610

**Published:** 2023-07-11

**Authors:** Yimeng Mao, Yuchen Zhao, Lingyun Zhang, Jie Li, Abu S Abdullah, Pinpin Zheng, Fan Wang

**Affiliations:** ^1^Fujian Provincial Center for Disease Control and Prevention, Fuzhou, Fujian, China; ^2^Department of Preventive Medicine and Health Education, School of Public Health, Institute of Health Communication, Key Lab of Public Health Safety of Ministry of Education, Fudan University, Shanghai, China; ^3^School of Journalism and Communication/National Media and Experimental Teaching Center, Jinan University, Guangzhou, China; ^4^Duke Global Health Institute, Duke University, Durham, NC, United States; ^5^Fudan Development Institute, Fudan University, Shanghai, China

**Keywords:** vaccine recommendation frequency, HPV vaccine, health care provider, knowledge of HPV, vaccine accessibility, communication difficulty

## Abstract

**Introduction:**

Human papillomavirus (HPV) infection, an important pathogenic factor for cervical cancer, can be prevented by the HPV vaccine. Health care provider (HCP) recommendations contribute to improve HPV vaccination coverage. The aim of this study was to assess the frequency of HCP recommendations for HPV vaccination and associated factors.

**Methods:**

From Nov 8 to Dec 6 in 2018, a cross-sectional study was conducted through online questionnaires among HCPs (*n* = 1,371) from hospitals in three large cities in China (Shanghai, Guangzhou, and Shenzhen). Data on demographic characteristics, the frequency of HPV vaccination recommendations, HPV knowledge and related attitudes were collected through the questionnaires.

**Results:**

Among 1,371 participants, only 30.2% reported that they frequently recommended HPV vaccination. Multivariate analyses indicated that female sex, being employed in obstetrics or gynecology departments and community health service centers, and having higher self-reported and actual knowledge of HPV were factors associated with a higher recommendation frequency. Factors including a self-perceived non-obligation to provide recommendations and difficulties in discussing sexual topics were significantly correlated with less frequent recommendations. Employment in a community health service center (OR = 2.068, 95% CI: 1.070–3.999) was the strongest factor associated with the frequency of HCPs’ recommendations for HPV vaccination.

**Discussion:**

The frequency of HCPs’ recommendations for HPV vaccination in China was much lower than that in many developed countries. To enhance the recommendation frequency, medical institutions should help HCPs gain more knowledge of HPV and master communication skills. At the same time, the government should take measures to enhance the accessibility of HPV vaccines. The media should help to alleviate people’s concerns and encourage them to face up sexual health.

## 1. Introduction

As the fourth most common cancer among women, cervical cancer is estimated to have caused 342,000 deaths globally and 59,060 deaths in China in 2020 (1, 2). Two types of human papillomavirus (HPV) (16 and 18) are responsible for almost 50% of high-grade cervical pre-cancers (1). To protect people from high-risk or oncogenic HPV infection, vaccination against HPV is the primary preventive comprehensive cervical cancer control measure in the global strategy (3). However, as of 2020, the estimated full-series cumulative HPV vaccination coverage rate was only 2.24% among females between 9 and 45 years old in China (4), while the global coverage rate of the final HPV dose was estimated at 15% (5). There seems to be a vast distance for HPV vaccination coverage in China to keep up with global coverage.

There have been great challenges in HPV vaccination since the vaccine was approved in mainland of China in 2016. First, as a preventive treatment, HPV vaccination was not covered by health insurance in most cases (6, 7). Additionally, the costs of HPV vaccines in China are high for the public (7), ranging from RMB 987 (2vHPV, Cecolin) to RMB 3910 (9vHPV, Gardasil®9), which raises questions about their cost-effectiveness (7, 8). Even so, HPV vaccine supplies are inadequate. Sometimes people have to wait for a long time, even up to 2 years, to make an online appointment for vaccination (7, 9). In addition, HPV vaccination has been culturally constructed as an indicator of females’ sexual morality to some extent in China. It is sometimes seen as encouragement for promiscuity and a necessity for those engaged in promiscuous sexual activities (8).

As opinion leaders on the topic of health, health care providers (HCPs) play an important role in HPV vaccination (10). Females who received a strong recommendation from an HCP were 4 times more likely to receive the HPV vaccine than those who received a weak recommendation (11). However, too strong HPV vaccine recommendation may also have negative effects on the patient vaccination rate. If HCPs show too much enthusiasm in their recommendations, patients might be suspicious of their motives, especially when the HPV vaccine is self-paid and expensive (8).

To date, although many studies have explored HCPs’ willingness to recommend HPV vaccines in China (12–14), the frequency of recommendation has rarely been considered. Compared with recommendation intentions, the actual recommendation frequency is a better measure of HCPs’ recommendation behaviors, reflecting the comprehensive influence of the environment, HCP abilities and doctor–patient communication.

Factors influencing HCPs’ recommendations are complicated. The existing studies in China have mainly focused on the impact of HCPs’ knowledge on recommendations (12–14). However, the influences of other factors, such as difficulty communicating with patients regarding sexual topics and whether HCPs realize that recommending vaccinations is their responsibility, remain unknown. Understanding and improving HCPs’ communication, particularly with regard to recommendations for HPV vaccination, has both theoretical and practical significance. Our study aimed to assess the frequency of HCPs’ recommendations for HPV vaccination and explore its related factors. These findings are expected to provide guidance for targeted measures that could enhance the frequency of HCPs’ recommendations for HPV vaccination, which is imperative to increasing HPV vaccination coverage in China.

## 2. Materials and methods

### 2.1. Study design and implementation

This multicenter cross-sectional study was conducted in Shanghai, Guangzhou, and Shenzhen, China. All of these cities are located in coastal areas with advanced economies and superior medical resources. The rapid development in Shanghai, as China’s economic and trade center, made new health policies and products more acceptable. Before the HPV vaccine was officially approved in mainland of China, HPV vaccination was already available in Hong Kong and Macao, which are geographically close to Shenzhen and Guangzhou. Thus, HCPs in Shanghai, Shenzhen and Guangzhou might have more communication regarding HPV and vaccination than those in other cities in China. The selection of these three cities could help us investigate more factors related to HCPs’ recommendations for HPV vaccination.

Purposive sampling was used in this study. We chose 13 third-class general hospitals in the three cities (4 in Shanghai, 6 in Shenzhen, and 3 in Guangzhou). In addition, we chose 4–6 districts in each city. Maternal and child hospitals in each district were invited to participate in our study. Community health centers in each city were purposively selected, with an average of approximately 10, covering the different geographic locations and representing the different economic levels of the city. In total, we selected 20 maternal and child health hospitals, 29 community health centers and 13 general hospitals.

Physicians and nurses who worked in obstetrics and gynecology, preventive health care, pediatrics and general medicine departments in the above institutions were mainly invited to the survey. Females between 9 and 45 years old were the target people for HPV vaccination. Consultation regarding HPV vaccination was mainly provided by gynecologists and pediatricians. Besides, HPV vaccine is usually provided by community health service centers, while preventive health care and general medicine departments were the important departments in HPV vaccination administration. Thus, we selected HCPs from these departments for they were more closely related to HPV vaccination.

So far, there is no similar research in China. Although there are more than a dozen foreign literatures on this topic, the results of other countries may not be applicable considering the unique social background of China. Therefore, the proportion of frequent vaccination recommendations in HCPs is estimated at 50% to achieve the most conservative sample size. Assuming an alpha error of 5%, an allowable error of 0.04, and a 70% response rate, the target minimum sample size is calculated to be 858. To ensure that the minimum sample size was reached, we planned to obtain at least 15 providers from each institution. For institutions with fewer than 15 HCPs, all eligible HCPs were invited to participate in the survey.

This study was conducted through online anonymous questionnaires by the Wenjuanxing platform (a popular online survey platform in China, available at: https://www.wjx.cn/app/survey.aspx) from November 8 to December 6 in 2018. Each participating hospital had a contact person. After receiving the online questionnaire link from the study team, the contact person sent the link to each participant. During the survey period, the HCPs who received the link could open the questionnaire and complete the survey. In total, 1,616 HCPs were invited for the survey, and 1,396 (86.4%) questionnaires were returned. Questionnaires were identified valid if they: (1) Took more than 2 min to complete the survey; (2) The question about common sense was answered correctly (Question: What is the capital of China? Correct answer: Beijing); (3) No obvious conflicts between the answers. After information sorting and cleaning, 25 (1.8%) participants were excluded in the current study. We used the STROBE cross sectional reporting guidelines to make sure that our study included all the items needed for survey reporting (Supplementary material 1) (15). This study was approved by the institutional review board of the School of Public Health, Fudan University (IRB#2018-04-0677-B).

### 2.2. Measures

There were totally 32 questions in the questionnaire, which could be divided into four sections including demographic information, frequency of HPV vaccine recommendation, knowledge of HPV and attitudes toward HPV-related topics (Supplementary material 2).

#### 2.2.1. Frequency of HPV vaccine recommendations

The frequency of HPV vaccine recommendations was measured by the following question: “How often have you recommended HPV vaccination for age-appropriate patients in the past 3 months?” The response options included (1) never, (2) seldom, (3) sometimes, (4) usually and (5) always. For our analyses, we dichotomized the responses, combining the “usually” and “always” responses (frequent recommendation of HPV vaccines) versus the combination of all other responses (infrequent recommendation of HPV vaccines) (16, 17).

#### 2.2.2. Covariates

Demographic information, knowledge of HPV and attitudes toward different HPV-related topics were measured as covariates. The demographic information included sex, age, occupation (physician, nurse), department (obstetrics and gynecology, preventive health care, pediatrics, general medicine and others), title (primary, intermediate, senior), hospital type (community health service center, maternal and child hospital, general hospital), and city (Shanghai, Guangzhou, Shenzhen).

Knowledge of HPV included actual and self-reported knowledge. The actual knowledge refers to one’s knowledge of HPV and HPV vaccine in actual. It was measured by 10 HPV-related questions; every question was multiple-choice, and there was only one correct answer. The actual knowledge score was calculated by summing the number of correct responses to the questions. The self-reported knowledge is how much the HCPs think themselves know about HPV, cervical cancer and HPV vaccine. We designed three questions to assess self-reported knowledge of HPV, cervical cancer and HPV vaccination. Detailed information on the survey questions is shown in the Supplementary material 2 (Section III).

Based on a literature review and considering the Chinese context and general views from a previous discussion with HCPs, we formulated eight items on attitudes toward different HPV-related topics. These topics encompass HCPs’ self-perceived lack of obligation to recommend vaccines, apprehensions about being perceived as pushy salespeople, beliefs that their patients face a low risk of HPV infection and related diseases, skepticism towards the HPV vaccine, and difficulty in discussing sensitive sexual topics. Detailed information on the related survey questions, variable descriptions and processing is shown in the Supplementary material 2 (Section IV).

#### 2.2.3. Concerns and difficulties in discussing HPV/sexual topics with patients

Difficulty in discussing HPV/sexual topics with patients or parents of young patients may decrease the frequency of HCPs’ recommendations for HPV vaccination. For HCPs who reported having discussions with patients or parents of young patients about HPV vaccines, two additional probing questions were asked regarding patients’ concerns about HPV vaccines and the difficulties in discussing sexual topics with parents of young patients (Supplementary material 2, Section V).

### 2.3. Statistical analysis

Frequencies and proportions are reported for HCPs’ HPV vaccine recommendations. The median and interquartile ranges (IQR) are reported for the knowledge of HPV and the attitudes toward different HPV-related topics. The Chi-square tests was used to explore the association between demographic characteristics and HPV recommendation frequency. Internal consistency reliability was assessed by calculating Cronbach’s α coefficient for self-reported knowledge of HPV, low risk of infection/disease and skepticism regarding the HPV vaccine. Multivariate logistic regression analyses were performed to assess the association between the potential influencing factors and the frequency of HPV vaccine recommendations after controlling for related characteristic covariates. Adjusted odds ratios (ORs) and their 95% confidence intervals (CIs) were used to quantify the effects. IBM SPSS software version 20.0 (SPSS, Inc., Chicago, Illinois, US) was used to carry out all analyses. All tests were two-sided, and *p* < 0.05 was considered statistically significant.

## 3. Results

### 3.1. Descriptive statistics

#### 3.1.1. Demographic characteristics and HPV vaccine recommendation frequency

Among 1,616 HCPs who were invited, 1,396 (86.4%) participated the survey with 1,371 (98.2%) valid questionnaires. Among those valid participants, 1,177 were female (85.8%), and 194 were male (14.2%), with an average age of 34.9 ± 8.0 years. The occupations of the participants were physicians (66.4%) and nurses (33.6%). Almost half of the participants (43.4%) worked in obstetrics and gynecology departments, and 17.9% worked in preventive health care departments. Most participants’ titles were primary (42.3%) and intermediate (46.2%). A total of 46.8% of the participants worked in maternal and child hospitals, and 35.6% worked in community health service centers (Table 1).

**Table 1:** Demographic information and the frequency of recommending HPV vaccination among participants.

Variables		N	%
Sex	Male	194	14.2
	Female	1,177	85.8
Age (years)	≤30	476	34.7
	31–40	597	43.5
	41–50	247	18
	>50	51	3.7
Occupation	Physician	910	66.4
	Nurse	461	33.6
Department	Pediatrics	199	14.5
	General medicine	273	19.9
	Obstetrics and gynecology	595	43.4
	Preventive health care	245	17.9
	Other	59	4.3
Title	Primary	580	42.3
	Intermediate	634	46.2
	Senior	157	11.5
Hospital type	Community health service center	488	35.6
	Maternal and child hospital	642	46.8
	General hospital	241	17.6
City	Shanghai	409	29.8
	Guangzhou	447	32.6
	Shenzhen	515	37.6
Frequency of recommendation	Always	97	7.1
	Usually	317	23.1
	Sometimes	496	36.2
	Seldom	320	23.3
	Never	141	10.3

The number of participants who never, seldom and sometimes recommended HPV vaccination to their patients was 141 (10.3%), 320 (23.3%) and 496 (36.2%), respectively, and all of them were categorized as infrequently recommending HPV vaccination (957, 69.8%). A total of 23.1% of the participants reported that they usually recommend HPV vaccines, and 7.1% reported always recommending HPV vaccines; all of them were categorized as frequently recommending HPV vaccines (414, 30.2%) (Table 1). The univariate analysis showed that gender, age, city of the HCPs, departments as well as their working titles were associated with the frequency of HPV vaccine recommendation (Table 2).

**Table 2:** Demographic characteristics associated with HPV vaccine recommendation frequency.

Variables		Infrequent recommendation N (%)	Frequent recommendation N (%)	*p*
Total		957 (69.8)	414 (30.2)	
Sex	Male	153 (78.9)	41 (21.1)	0.003
	Female	804 (68.3)	373 (31.7)	
Age (years)	≤30	366 (76.9)	110 (23.1)	<0.001
	31–40	406 (68.0)	191 (32.0)	
	41–50	154 (62.3)	93 (37.7)	
	>50	31 (60.8)	20 (39.2)	
Occupation	Physician	625 (68.7)	285 (31.3)	0.204
	Nurse	332 (72.0)	129 (28.0)	
Department	Pediatrics	172 (86.4)	27 (13.6)	<0.001
	General medicine	200 (73.3)	73 (26.7)	
	Obstetrics and gynecology	385 (64.7)	210 (35.3)	
	Preventive health care	155 (63.3)	90 (36.7)	
	Other	45 (76.3)	14 (23.7)	
Title	Primary	438 (75.5)	142 (24.5)	<0.001
	Intermediate	424 (66.9)	210 (33.1)	
	Senior	95 (60.5)	62 (39.5)	
Hospital type	Community health service center	336 (68.9)	152 (31.1)	0.250
	Maternal and child hospital	442 (68.8)	200 (31.2)	
	General hospital	179 (74.3)	62 (25.7)	
City	Shanghai	291 (71.1)	118 (28.9)	0.002
	Guangzhou	334 (74.7)	113 (25.3)	
	Shenzhen	332 (64.5)	183 (35.5)	

#### 3.1.2. Knowledge of HPV

For the knowledge of HPV, the actual HPV knowledge score ranged from 0 to 10 points, with a median score of 8.00 (IQR: 7.00–9.00). The self-reported knowledge of HPV score ranged from 1 to 5 points, with a median score of 3.33 (IQR: 2.67–4.00). The Cronbach’s α coefficient of the self-reported knowledge of HPV was 0.92. The description of the rate of each answer for actual and self-reported HPV knowledge was as following (Table 3).

**Table 3:** Description of the variables for actual and self-reported HPV knowledge.

	Questions	Variable description^a^	N^b^	%
Actual	(1) What is the main infection route of HPV infection?	Sexual contact	824	60.1
	(2) Which age group is at the highest risk of becoming infected with HPV?	15–35 years	1,117	81.5
	(3) Which sex is likely to be screened for HPV?	Male and female	870	63.5
	(4) Which two HPV types have been shown to cause 70% of cervical cancers?	16 and 18	1,084	79.1
	(5) When the number of sexual partners increases, does the risk of HPV infection increase?	Yes	1,268	92.5
	(6) Which disease is associated with HPV 16 and 18?	Cervical cancer	1,192	86.9
	(7) Which population is mainly targeted for HPV vaccination?	Uninfected people	1,349	98.4
	(8) If a patient becomes infected with HPV, does the HPV vaccine provide protection?	Yes	837	61.1
	(9) After HPV vaccination, is cervical cancer screening still necessary?	Yes	1,192	86.9
	(10) What is the age range for women to receive a preventive 4vHPV vaccine?	20–45 years	893	65.1
Self-reported	(1) How much do you know about HPV?	1 = Totally unknown2 = Unknown 3 = Neutral4 = Known 5 = Totally known	713	52.0
(Cronbach’s α = 0.92)	(2) How much do you know about cervical cancer?		753	54.9
	(3) How much do you know about HPV vaccines?		631	46.0

a Only the correct answers to questions regarding “actual knowledge” are shown.

b In the section “actual knowledge,” the number of participants who answered correctly is shown. In the section “Self-reported knowledge,” the number of participants who chose “Known” or “Totally known” is shown.

#### 3.1.3. Attitudes toward different HPV-related topics

Apart from difficulty in communicating regarding sexual topics (scores ranging from 1 to 4), the scores of the other variables ranged from 1 to 5 (Table 4). The median score of perceived non-obligation to recommend vaccines was 3.00 (IQR: 2.00–3.00); the median score of concern over being seen as a hard seller was 3.00 (IQR: 3.00–4.00); and the median score for low risk of infection/disease was 2.50 (IQR: 2.00–3.00). Skepticism regarding the HPV vaccine had a median score of 3.00 (IQR: 2.67–3.33). The median score of difficulty in discussing sexual topics was 2.00 (IQR: 1.00–3.00). The Cronbach’s α coefficient of both of low risk of infection/disease and skepticism regarding the HPV vaccine was higher than 0.60.

**Table 4:** Description of Attitudes toward HPV-related topics.

Variable	Indicators	Variable description	N^a^	%
Self-perceived non-obligation to recommend vaccines	I have no obligation to talk to my patients about HPV.	1 = Totally disagree 2 = disagree 3 = Neutral 4 = Agree 5 = Totally agree	309	22.5
Concern over being seen as a hard seller	I do not want to look like I’m trying to sell an expensive vaccine.		637	46.5
Low risk of infection/disease ^a^	(1)Most of my patients are not at risk for cervical cancer		300	21.9
(Cronbach’s α = 0.73)	(2)Most of my patients are not at risk for HPV infection		186	13.6
Skepticism regarding the HPV vaccine ^a^	(1)The effectiveness of the HPV vaccine is uncertain		702	51.2
(Cronbach’s α = 0.61)	(2)The safety of the HPV vaccine is uncertain		514	37.5
	(3)The HPV vaccine is not cost-effective		186	13.6
Difficulty in discussing sexual topics	Do you think it is difficult to talk about sex and HPV vaccination with female patients?	1 = Very easy 2 = Easy 3 = Difficult 4 = Very difficult	507	37.0

a The number of participants who chose “Agree” or “Totally agree” (“Difficult” or “Very difficult”) is shown.

### 3.2. Association of the frequency of HPV vaccine recommendations with influencing factors

With infrequent HPV vaccination recommendation as a reference, logistic regression analyses revealed the following (Table 5): Comparing to male HCPs, female HCPs were more likely to recommend HPV vaccines to their patients (OR = 1.643, 95% CI: 1.072–2.518); HCPs working in obstetrics and gynecology departments (OR = 1.655, 95% CI: 1.013–2.705) recommended HPV vaccination more frequently than those working in pediatric departments. Compared to general hospitals, in community health service centers, the intention to frequently recommend HPV vaccination was higher (OR = 2.068, 95% CI: 1.070–3.999). HCPs who worked in Shenzhen were more likely to recommend HPV vaccine than HCPs in Shanghai (OR = 1.560, 95% CI: 1.119–2.175).

**Table 5:** Logistic regression of HPV vaccination recommendations from health care providers.

Block	Variable	Β	*P*	OR	95% CI
Block 1	Demographic characteristics				
(*R*^2^ = 0.095)	**Age** (Ref: ≤30)				
	31–40	0.059	0.771	1.061	0.714–1.576
	41–50	0.331	0.190	1.393	0.848–2.286
	>50	0.475	0.235	1.608	0.734–3.523
	**Sex** (Ref: Male)				
	Female	**0.497**	**0.023**	**1.643**	**1.072–2.518**
	**Occupation** (Ref: Physician)				
	Nurse	−0.055	0.72	0.946	0.700–1.279
	**Department** (Ref: Pediatrics)				
	General Medicine	0.084	0.832	1.088	0.501–2.361
	Obstetrics and gynecology	**0.504**	**0.044**	**1.655**	**1.013–2.705**
	Preventive health care	0.584	0.096	1.794	0.902–3.568
	Other	0.121	0.776	1.128	0.492–2.589
	**Title** (Ref: Primary)				
	Intermediate	0.228	0.233	1.256	0.864–1.825
	Senior	0.103	0.716	1.108	0.637–1.929
	**Hospital type** (Ref: General hospitals)				
	Community health service centers	**0.727**	**0.031**	**2.068**	**1.070–3.999**
	Maternal and child hospitals	0.276	0.162	1.318	0.895–1.942
	**City** (Ref: Shanghai)				
	Guangzhou	0.000	0.998	1.000	0.710–1.407
	Shenzhen	**0.445**	**0.009**	**1.560**	**1.119–2.175**

Block 2	Knowledge of HPV				
(*R*^2^ = 0.090)	Self-reported knowledge of HPV	**0.588**	**0.000**	**1.800**	**1.539–2.106**
	Actual knowledge of HPV	**0.126**	**0.008**	**1.134**	**1.033–1.245**

Block 3	Attitudes toward HPV-related topics				
(*R*^2^ = 0.050)	Self-perceived non-obligation to recommend	**−0.260**	**0.001**	**0.771**	**0.659–0.901**
	Concern over being seen as a hard seller	−0.099	0.138	0.905	0.794–1.032
	A low risk of infection/disease in patients^a^	0.127	0.182	1.135	0.942–1.368
	Skepticism regarding the HPV vaccine ^a^	0.195	0.082	1.216	0.975–1.515
	Difficulty in discussing sexual topics	**−0.466**	**0.000**	**0.628**	**0.535–0.736**
	Constant	−3.941	0.000	0.019	

a The average score of the questions in “a low risk of infection/disease” and “skepticism regarding the HPV vaccine” were calculated and included in the logistics regression model. The bold means that the variable is statistically significant in its association with the HPV vaccination recommendations.

Knowledge and attitude of HCPs also influenced the frequency of recommendations. Participants who had higher self-reported and actual knowledge of HPV recommended HPV vaccination more frequently (OR = 1.800, 95% CI: 1.539–2.106; OR = 1.134, 95% CI: 1.033–1.245). In contrast, for HCPs who perceived no obligation to recommend vaccination (OR = 0.771, 95% CI: 0.659–0.901) or experienced difficulty in discussing sexual topics with their patients (OR = 0.628, 95% CI: 0.535–0.736), the frequency of recommendation decreased. The total Nagelkerke R^2^ of this logistic regression was 0.235.

### 3.3. Concerns and difficulties in discussing HPV/sexual topics with patients

Many HCPs thought that their patients worried about the efficacy (37.5%) and safety (32.5%) of the HPV vaccine. In addition, 13.7% of the participants claimed that it was difficult for patients to receive HPV vaccines. The majority of participants found that parents of young patients were unwilling to start a sex-related conversation with their children (63.5%). A total of 38.0% of the participants thought that parents regarded sexually transmitted diseases (STDs) as rarely occurring in children. More detailed information was listed in Table 6.

**Table 6:** Concerns and difficulties in discussing HPV/sexual topics with patients.

Variables	*N*	%
Patients’ concerns about HPV vaccines		
The efficacy of HPV vaccines	465	37.5
The safety of HPV vaccines	403	32.5
The accessibility of HPV vaccines	170	13.7
The applicable age of HPV vaccines	84	6.8
The price of HPV vaccines	64	5.2
The benefit of HPV vaccination	53	4.3
Patients’ difficulties in discussing sexual topics		
Unwillingness to start a sex-related conversation with children	807	63.5
An STD in a child is rare	483	38.0
Vaccination is a social stigma associated with STDs	281	22.1
Vaccination may lead to premature sexual behaviors in children	251	19.8
Children may think they are protected and engage in sexual activities after vaccination	227	17.9

## 4. Discussion

This study is among the first studies that evaluated the frequency of HCPs’ recommendations for HPV vaccination in China. Moreover, we verified the association of HCPs’ knowledge of HPV and attitudes toward various HPV-related topics with their recommendations for HPV vaccination. All of the above findings provided in-depth evidence explaining the low prevalence of frequent recommendations for HPV vaccination. The findings also provide theoretical evidence for exploring approaches to enhance the frequency of HPV vaccine recommendations by Chinese HCPs and achieve high HPV vaccination coverage to realize the goal of “zero” cervical cancer cases.

In this study, less than one-third of Chinese HCPs frequently recommended HPV vaccination, which is much lower than the recommendation (always or usually) made by HCPs in the United States (79.0%) and France (72.4%) (16, 17). With HPV vaccines having been approved for more than 10 years, the low frequency of HCPs’ recommendations in China is a cause for public health concern. However, in a prior national survey with only two answer options (willing and unwilling), 94.8% of the Chinese HCPs claimed that they were willing to recommend HPV vaccination to their patients (14), indicating the large gap between willingness and actual recommendations which further highlights the importance of exploring factors that influence recommendations.

According to our findings, female HCPs were more likely than male HCPs to frequently recommend HPV vaccination in China. Because female HCPs may pay more attention to cervical cancer as a women’s health issue, they may be more flexible in dealing with sexual health issues (18, 19). In terms of departments, the frequency of recommendations made by obstetrician-gynecologists is higher than that of pediatricians because obstetrician-gynecologists not only know the potential health effects of HPV infection but also take responsibility for offering primary health care services for women of childbearing age (20, 21). Ensuring frequent recommendations by both obstetricians-gynecologists and pediatricians is equally essential, as girls aged between 9 and 15 years old are an important target population for HPV vaccination. To achieve a higher frequency of HPV vaccination recommendations, more attention should also be paid to male HCPs, especially those who work in pediatric departments.

Knowledge of HPV played a key role in the low frequency of HPV vaccine recommendations (12, 22), which was also the most powerful block in our logistic regression model. Both actual and self-reported HPV knowledge could enhance the recommendation intentions of HCPs (12, 23–25). The actual knowledge of HPV reflected whether an HCP knew the risks of HPV infection and the protective effect of the HPV vaccine. In a previous study, the low knowledge level was the primary reason for unwillingness to recommend HPV vaccination in Western China (12). The self-reported knowledge reflected self-efficacy that HCPs believe themselves having enough knowledge to discuss with their patients about HPV vaccine (26). In addition, HCPs with higher self-reported knowledge could communicate the benefits of the HPV vaccine clearly and confidently and address their patients’ doubts regarding the HPV vaccine (27). Thus, it is necessary to provide HCPs with HPV-related training to improve not only their actual knowledge but also their self-reported knowledge, which is essential to increase their self-efficacy in discussing relevant topics with patients.

According to the WHO strategy, every HCP has the duty to ensure that women and adolescents receive the health services they need, including HPV vaccination (3). It is an HCP’s obligation to recommend the HPV vaccine to their patients (28, 29). However, almost one quarter (22.5%) of the HCPs still agreed with the viewpoint that “they had no obligation to talk to their patients about HPV.” Consultations with doctors in China mainly involved discussions regarding disease and treatment methods rather than preventive measures (8). This could partly be attributed to the lack of preventive medicine knowledge in clinical education (30). Previous studies indicated that the proportion of preventive medicine curricula, such as HPV vaccines, was insufficient in current clinical medicine training programs, which led to HCPs’ poor awareness of disease prevention (30, 31).

According to HCPs, one of the concerns patients have about HPV vaccines in China is the accessibility of vaccines. Because Shenzhen is the nearest city to Hong Kong, it is convenient for people in Shenzhen to go to Hong Kong to get HPV vaccination. Thus, HCPs in Shenzhen were more likely to recommend vaccine. As one important source of HPV vaccine supplements, the frequency of HCPs’ recommendations in community health service centers was higher than that of HCPs in general hospitals. Only HCPs in preventive health care department were permitted to prescribe the vaccine for their patients in China (32). The vaccine supply was derailed between general medicine and vaccination departments. In addition, approximately half of the participants stated that they did not want to seem like a hard seller of HPV vaccines, especially when the costs of vaccines were very high. Patients have to wait for a long time and pay a high price for the HPV vaccine (7, 33). The low frequency of HCPs’ recommendations may be partly due to the time and financial cost of the vaccine.

In view of the sex symbolic meaning of the HPV vaccine, difficulty in communication with patients regarding sexual topics is also a barrier (8). Sexuality is a sensitive and private topic related to moral and culture in China, whereas traditionally, people can only obtain limited education related to sex from family members and school (34, 35). Individuals from conservative social backgrounds often perceive sex as shameful or guilt-inducing, leading them to avoid open discussions on this topic (36). When talking about sex with someone from a conservative social background, especially children and their parents, HCPs may fear offending their patients and avoid talking about HPV-related topics (24, 25, 36, 37). The “unwillingness to start a sex-related discussion with children” and “vaccination is a social stigma associated with STDs” became the main reasons for the difficulties in discussing sexual topics with parents of young patients. Furthermore, HCPs may assume that young people from a conservative social background are less likely to engage in high-risk sexual behaviors and therefore have a lower risk of HPV exposure (24). Thus, they may think that it is not necessary to recommend HPV vaccination (38). In fact, the number of people who have high-risk sexual behaviors such as multiple sexual partners is increasing and their age is getting younger in China (39). This underscores the fact that minors are also at high risk of HPV infection (39–41).

The frequency of HCPs’ recommendations played an important role in HPV vaccination coverage. First, medical institutions should hold regular training to increase HCPs’ knowledge of HPV and help them realize their responsibility in practicing clinical preventive medicine, including recommending HPV vaccination to patients. The preventive medicine curriculum in clinical medicine training programs needs to be strengthened to promote the concept of putting prevention first and the integrated development of preventive medicine and clinical medicine (30, 31).

To cope with the difficulties in communicating with patients regarding HPV/sexual topics, the “provider-driven” and “bundling” recommendation styles were suggested by the Centers for Disease Control and Prevention (CDC) and the American Academy of Family Physicians (AAFP), respectively (42–44). These recommendation styles require the HCPs to inform patients that they should receive the HPV vaccine at an appropriate age and bundle the HPV vaccine with routine immunization (45). In addition, regardless of prior exposure to HPV and sexual activity, HCPs should be reminded to provide recommendations for all patients of appropriate age, not just those perceived to be “at risk” (29, 44).

To enhance the accessibility of HPV vaccines, the range of institutions that have the right to supply HPV vaccines should be expanded, not just community health centers. The American College of Obstetricians and Gynecologists (ACOG) suggested that the HPV vaccine could be stocked and administered by clinicians in clinics when feasible (29). This will make HCPs take part in vaccination deeply, not just as advisors. For children, a school-based HPV vaccine recommendation system with the cooperation of HCPs may be suitable and effective (46). In addition, expanding access to HPV vaccination is also essential. Possible solutions include single-dose vaccination, low-cost domestic vaccines, and co-administration with other vaccines for adolescents (47). Whether the domestic HPV vaccine should be included in the publicly funded Expanded Program on Immunization (EPI) requires more cost-effectiveness tests in China (33).

The media plays an important role in recommending HPV vaccination to the public. More information about the HPV and HPV vaccine should be delivered to the public through media. The effectiveness and safety of HPV vaccines have become the greatest concerns of patients. The media could track and provide real-time data about the effectiveness and safety of HPV vaccines to alleviate the concerns of the public. Moreover, the media should also call for people to face up sexual health topics and promote correct sexual health education, especially for those from a conservative social background and those having active sexual behaviors (48), which could help reduce the difficulties for HCPs in communication regarding sexual topics.

There are still a few limitations in this study. First, as a cross-sectional study, the causal relationship between the frequency of recommendations and associated factors remains uncertain. Second, the participants in this study were mostly from developed cities within China which is different from other cities in China and other countries in terms of cultures and health policy. The sample may not be representative of HCPs throughout China, and the results may overestimate the frequency of recommendations. Furthermore, all data collected in this study were self-reported and the HPV recommendation frequency was measured in subjective terms which may generate information bias. Further research may use longitudinal approaches and more objective measures across the country to explore factors influencing the frequency of recommendations in China.

## 5. Conclusion

In conclusion, our study showed that the frequency of HCPs’ recommendations for HPV vaccination is relatively low in China. This could be partly attributed to the lack of HCPs’ knowledge of HPV and HPV vaccine. The difficulty in discussing sexual topics and the sense of having no obligation to recommend vaccines are also barriers to HCPs’ recommendations. It is essential to enhance HCPs’ knowledge of HPV and help them master the skills to communicate with patients about sexual health and HPV vaccination. The government should take measures to enhance the accessibility of HPV vaccines. The media should provide accurate information about the vaccines to alleviate people’s concerns and encourage them to face up sexual health topics.

## Data availability statement

The raw data supporting the conclusions of this article will be made available by the authors, without undue reservation.

## Ethics statement

This study was approved by the institutional review board of the School of Public Health, Fudan University (IRB#2018-04-0677-B).

## Author contributions

PZ and FW designed and organized the study. PZ, FW, YM, and LZ performed the survey. YZ and YM undertook the data analysis and interpretation, supervised by PZ, and wrote the manuscript. PZ, FW, JL, and AA reviewed and commented on the manuscript. All authors have read and agreed to this version of the manuscript.

## Funding

This research was funded by Project HOPE of the “Cervical Cancer/HPV Prevention Education Program” and the project “Advocacy Activities Design to Improve Health Literacy of New Media among Shanghai Teenagers,” commissioned by the Shanghai Audio-Visual Education Museum.

## Conflict of interest

The authors declare that the research was conducted in the absence of any commercial or financial relationships that could be construed as a potential conflict of interest.

## Publisher’s note

All claims expressed in this article are solely those of the authors and do not necessarily represent those of their affiliated organizations, or those of the publisher, the editors and the reviewers. Any product that may be evaluated in this article, or claim that may be made by its manufacturer, is not guaranteed or endorsed by the publisher.
