# Supplementary material for: Frequency of health care provider recommendations for HPV vaccination: a survey in three large cities in China
Source: Front Public Health. 2023 Jul 11;11:1203610. doi: 10.3389/fpubh.2023.1203610 (PMC10366465; doi:10.3389/fpubh.2023.1203610)
Supplement: Supplementary file 2 [file Table_2.DOCX]

Supplementary Material 2

The complete questionnaire for investigation

**Section I: Demographic information.**

| 1. The sex A. Male B. Female |
| --- |
| 2. The age (gap filling) |
| 3. The occupation A. Physician B. Nurse |
| 4. The department in hospital  A. Pediatrics B. General medicine C. Obstetrics and gynecology  D. Preventive health care E. Other |
| 5. The title A. Primary B. Intermediate C. Senior |
| 6. The hospital type  A. Community health service center B. Maternal and child hospitals C. General hospitals |
| 7. Your city A. Shanghai B. Guangzhou C. Shenzhen |
| 8. What’s the capital of China? (Question of common knowledge)  A. Tianjin B. Beijing C. Nanjing |

**Section II: Frequency of recommendation**

| 9. How often have you recommended HPV vaccination for age-appropriate patients in the past 3 months?  A. Always B. Usually C. Sometimes D. Seldom E. Never |
| --- |

**Section III: Knowledge of HPV**

There are only one correct answer in each of following 10 questions, please choose the one you think is correct.

| 10. What is the main infection route of HPV infection?  A. Direct contact B. Indirect contact C. Sexual contact D. All of the above |
| --- |
| 11. Which age group is at the highest risk of becoming infected with HPV?  A. 0-14 years B. 15-35 years C. ＞35 years |
| 12. Which sex is likely to be screened for HPV?  A. Male B. Female C. Male and female D. Unclear |
| 13. Which two HPV types have been shown to cause 70% of cervical cancers?  A. 16 & 33 B. 16 & 18 C. 42 & 51 D. 18 & 42 |
| 14. When the number of sexual partners increase, does the risk of HPV infection increase?  A. Yes B. No C. Unclear |
| 15. Which disease is associated with HPV 16 and 18?  A. Genital warts B. Plantar warts C. Cervical cancer D. Unclear |
| 16. Which population is mainly targeted for HPV vaccination?  A. Uninfected people B. Infected people |
| 17. If a patient becomes infected with HPV, does the HPV vaccine provide protection?  A. Yes B. No C. Unclear |
| 18. After HPV vaccination, is cervical cancer screening still necessary?  A. Yes B. No C. Unclear |
| 19. What is the age range for women to receive a preventive 4vHPV vaccine?  A. 9-25 years B. 20-45 years C. 9-26 years D. 15-30 years E. Unclear |

Please choose the one you think is most practical for yourself.

| 20. How much do you know about HPV?  A. Totally unknown B. Unknown C. Neutral D. Known E. Totally known |
| --- |
| 21. How much do you know about cervical cancer?  A. Totally unknown B. Unknown C. Neutral D. Known E. Totally known |
| 22. How much do you know about HPV vaccines?  A. Totally unknown B. Unknown C. Neutral D. Known E. Totally known |

**Section IV: Attitudes toward HPV-related topics**

Please choose the one you think is most practical for yourself.

| 23. I have no obligation to talk to my patients about HPV.  A. Totally disagree B. Disagree C. Neutral D. Agree E. Totally agree |
| --- |
| 24. I don't want to look like I'm trying to sell an expensive vaccine.  A. Totally disagree B. Disagree C. Neutral D. Agree E. Totally agree |
| 25. Most of my patients are not at risk for cervical cancer  A. Totally disagree B. Disagree C. Neutral D. Agree E. Totally agree |
| 26. Most of my patients are not at risk for HPV infection.  A. Totally disagree B. Disagree C. Neutral D. Agree E. Totally agree |
| 27. The effectiveness of the HPV vaccine is uncertain.  A. Totally disagree B. Disagree C. Neutral D. Agree E. Totally agree |
| 28. The safety of the HPV vaccine is uncertain.  A. Totally disagree B. Disagree C. Neutral D. Agree E. Totally agree |
| 29. The HPV vaccine is not cost-effective  A. Totally disagree B. Disagree C. Neutral D. Agree E. Totally agree |
| 30. Do you think it is difficult to talk about sex with female patients for HPV vaccination?  A. Very easy B. Easy C. Difficult D. Very difficult |

**Section V: The concerns and difficulties in HPV/sexual topics discussion from patients**

| 31. When talking about the HPV, what are patients concerning about? (Multiple choice)  A. The efficacy of HPV vaccines  B. The safety of HPV vaccines  C. The accessibility of HPV vaccines  D. The applicable age of HPV vaccines  E. The price of HPV vaccines  F. The benefit of HPV vaccination |
| --- |
| 32. When talking about sexual topics with patients, what are the difficulties? (Multiple choice)  A. Unwillingness to start a sex-related conversation with children.  B. A sexually transmitted disease (STD) in a child is rare.  C. Vaccination is a social stigma associated with STD.  D. Vaccination may lead to children’ premature sexual behaviors.  E. Children may think themselves under protection and engage in sexual activities after vaccination. |
